# Studies in the *Stypella vermiformis* group (Auriculariales, Basidiomycota)

**DOI:** 10.1007/s10482-018-01209-9

**Published:** 2018-12-08

**Authors:** Viacheslav Spirin, Vera Malysheva, Danny Haelewaters, Karl-Henrik Larsson

**Affiliations:** 1 Botany Unit (Mycology), Finnish Museum of Natural History, University of Helsinki, P.O. Box 7, 00014 Helsinki, Finland; 2 Natural History Museum, University of Oslo, P.O. Box 1172, Blindern, 0318 Oslo, Norway; 3 Komarov Botanical Institute, Russian Academy of Sciences, Prof. Popova Str. 2, St. Petersburg, Russia 197376; 4 Department of Botany and Plant Pathology, Purdue University, 915 W. State Street, West Lafayette, IN 47907 USA

**Keywords:** 5 new taxa, 4 new typifications, Heterobasidiomycetes, Phylogeny

## Abstract

*Stypella vermiformis* is a heterobasidiomycete producing minute gelatinous basidiocarps on rotten wood of conifers in the Northern Hemisphere. In the current literature, *Stypella papillata*, the genus type of *Stypella* (described from Brazil), is treated as a taxonomic synonym of *S. vermiformis*. In the present paper, we revise the type material of *S. papillata* and a number of specimens addressed to *S. vermiformis*. As a result, the presumed synonymy of *S. papillata* and *S. vermiformis* is rejected and the genus *Stypella* is restricted to the single species *S. papillata*. Morphological and molecular phylogenetic studies of specimens from the Northern Hemisphere corresponding to the current concept of *S. vermiformis* uncovered three species from two newly described genera. *S. vermiformis* s.str. is distributed in temperate Europe and has small-sized basidia and basidiospores, and it is placed in a new genus, *Mycostilla*. Another genus, *Stypellopsis*, is created for two other species, the North American *Stypellopsis farlowii*, comb. nov., and the North European *Stypellopsis hyperborea*, sp. nov. Basidia and basidiospores of *Stypellopsis* spp. are larger than in *Mycostilla vermiformis* but other morphological characters are very similar. In addition, *Spiculogloea minuta* (Spiculogloeomycetes, Pucciniomycotina) is reported as new to Norway, parasitising basidiocarps of *M. vermiformis* and *Tulasnella* spp.

## Introduction

The Heterobasidiomycetes are an artificial group of fungi encompassing basidiomycetous taxa with septate basidia and (or) repetitive basidiospores (Weiss et al. 2004a). Their traditional division was based on features of basidial septation and sterigmata (Tulasne and Tulasne 1873) and survived almost unchanged for over a century. First studies on cell ultrastructure (Bandoni 1984) and then DNA studies (Fell et al. 2000; Weiss and Oberwinkler 2001; Bauer et al. 2006) questioned the reliability of these morphological characters for a higher-level taxonomy of basidiomycetes. Currently, the class and order arrangement of heterobasidiomycetes is more or less well-established (summarized in McLaughlin and Spatafora 2014). However, their lower-level taxonomy remains unstable, often due to the lack of sequence data or reference material (Liu et al. 2015; Wang et al. 2015). In many cases, this is also a result of a high morphological similarity of taxa appearing unrelated or only distantly related in phylogenetic studies (Millanes et al. 2011; Liu et al. 2015; Wang et al. 2015). An utmost case of this similarity is dealt with in the present paper.

The genus *Stypella* was described by Möller (1895) from Brazil for two newly introduced species with four-celled basidia, *Stypella papillata* and *Stypella minor*. It was not in use until Donk (1958, 1966) accepted it and selected *S. papillata* as the generic type. Next, Martin (1934, 1952) synonymized the European species *Heterochaetella crystallina* Bourdot with *S. papillata*. It was shown later that *Dacrymyces vermiformis* Berk. & Broome was an older name for *H. crystallina* and *S. papillata* as they were understood at that time (Reid 1974). Therefore, Reid (1974) created a new combination, *Stypella vermiformis* (Berk. & Broome) D.A. Reid, and placed both *H. crystallina* and *S. papillata* among its synonyms. This opinion about identity of *S. vermiformis* has been widely accepted in the modern taxonomy of heterobasidiomycetes (Oberwinkler 1982; Reid 1990; Roberts 1998).

In the protologues of *S. papillata* and *S. minor*, Möller (1895: 76) described and illustrated peculiar basidia; in his interpretation, each basidium is a terminal part of a much narrower hyphal segment. Studies of basidial morphology in *Exidia nucleata* (Schwein.) Burt showed that the hypha-like segment bearing four terminal basidial cells represents an integral part of the basidium in that species—the so-called ‘enucleate stalk’ (Wells 1964). Various opinions on the taxonomic value of this feature persist in the mycological literature. In particular, Donk (1966) accepted it as an important generic character although he refused to include all taxa with stalked basidia into one genus and considered them as belonging to four genera, *Myxarium*, *Stypella*, *Protodontia* and *Heterochaetella* (with a few species among *incertae sedis*). On the contrary, Roberts (1998) placed all species with effused basidiocarps and stalked basidia into a re-defined *Stypella,* whereas he retained the type species of *Myxarium*, *M. nucleatum* Wallr., in *Exidia*. However, none of these authors studied the authentic material of *S. papillata*.

Recent molecular phylogenetic studies (Weiss and Oberwinkler 2001; Wells et al. 2004; Spirin et al. 2017) mostly confirm the generic splitting advocated by Donk (1966). Therefore, the genus *Stypella* has been limited to the single species, *S. vermiformis* (= *S. papillata* sensu auct.). At the same time, stalked basidia are observed in some other genera of *Auriculariales* (Wells and Raitviir 1980; Weiss and Oberwinkler 2001; Malysheva and Spirin 2017). Thus, the taxonomic significance of this character may have been overemphasized. In the present paper, material of *Stypella vermiformis* from Northern Hemisphere is revised based on morphology and DNA data, and new information about *S. papillata* is provided.

## Materials and methods

### Morphological study

Collections and type specimens from several herbaria have been studied: University of Helsinki, Finland (H); Botanical Museum of the University of Oslo, Norway (O); University of Hamburg, Germany (HBG); University of Gothenburg, Sweden (GB); Farlow Herbarium at Harvard University, USA (FH); National Museum of Natural History, France (PC), and the private herbarium of Heikki Kotiranta in Helsinki, Finland (H.K.). Herbarium acronyms are given according to Thiers (2018). Morphological study follows Miettinen et al. (2012). The abbreviations used in microscopic descriptions are: L—mean basidiospore length, W—mean basidiospore width, Q—mean L/W ratio, n—number of basidiospore measurements per specimens studied.

### DNA extraction and sequencing

For DNA extraction, small fragments of dried basidiocarps were used. Extractions were done using the NucleoSpin Plant II Kit (Macherey–Nagel GmbH and Co. KG, Düren, Germany) following the manufacturer’s instructions. PCR amplification and sequencing of the nrITS region was performed using primers ITS1F (Gardes and Bruns 1993) and ITS4 (White et al. 1990). Primers JS1 (Landvik 1996) and LR5 (Vilgalys and Hester 1990) were used to amplify and sequence approximately 700 bp of nrLSU region. Sequencing was performed with an ABI model 3130 Genetic Analyzer (Applied Biosystems, CA, USA). Raw data were edited and assembled in MEGA 6 (Tamura et al. 2013).

### Phylogenetic analyses

For this study, eight nrITS and seven nrLSU sequences were generated. In addition to the sequences published here, 4 nrITS sequences and 43 nrLSU sequences were retrieved from GenBank (www.ncbi.nlm.nih.gov/genbank/). Sequences were aligned with the MAFFT version 7 web tool (http://mafft.cbrc.jp/alignment/server/) using the Q-INS-i option for nrITS and nrLSU. Before the phylogenetic analyses, the best-fit substitution models for the alignments (GTR) were estimated based on the Akaike Information Criterion (AIC) using FindModel web server (http://hiv.lanl.gov/content/sequence/findmodel/findmodel.html).

Two different phylogenetic analyses were performed for the nrLSU dataset: (1) Maximum likelihood (ML) analyses were run on the PhyML server v.3.0 (Guindon et al. 2010), with 100 rapid bootstrap (BS) replicates; (2) Bayesian inference analyses (BI) were run using MrBayes 3.2.5 software (Ronquist and Huelsenbeck 2003) for 5 million generations, under a GTR model, with four chains, and trees sampled every 100 generations. To check for convergence of MCMC analyses and to get estimates of the posterior distribution of parameter values, Tracer v1.6 was used (Rambaut et al. 2014). In total, 100,002 trees were read. Credible sets of trees contained 30,093 trees sampled. Burn-in was 1000 iterations. We accepted the result where the ESS (Effective Sample Size) was above 200 and the PSRF (Potential Scale Reduction Factor) was close to 1. For the nrITS dataset, we only performed ML analyses, again on the PhyML server v.3.0. Newly generated sequences have been deposited in GenBank (Table 1).

**Table 1:** Data for ITS and nrLSU sequences of *Mycostilla/Stypellopsis* spp. used in the phylogenetic analyses

Species	Collector/herbarium number	Origin (ISO code)	Host	nrLSU GenBank #	ITS GenBank #	Source
*M. vermiformis*	MW 417	DE	–	AF291369	–	Weiss and Oberwinkler (2001)
*M. vermiformis*	–	US-CA	indoor surface swab	–	KF221899	Adams et al. (2013)
*M. vermiformis*	–	SE	*Picea abies*	–	KM493985	GenBank
*M. vermiformis*	–	SE	soil	–	KU061904	GenBank
*M. vermiformis*	Oldervik 135.04 (O F188059)	NO	*Pinus sylvestris*	–	MG735418	Malysheva et al. (2018)
*M. vermiformis*	Spirin 11330 (H)	RU-NIZ	*P. abies*	MG735425	MG735417	Malysheva et al. (2018)
*M. vermiformis*	Spirin 11621 (O)	NO	*P. sylvestris*	MG857098	MG857093	this study
*S. farlowii*	Larsson 12337 (GB)	US-TN	decayed wood	MG857099	MG857095	this study
*S. farlowii*	Spirin 8254 (H)	US-WA	*Tsuga heterophylla*	MG857100	MG857094	this study
*S. hyperborea*	Larsson 11751 (GB)	FI	coniferous wood	EU118672	EU118672	GenBank
*S. hyperborea*	J. Nordén 9751 (O)	NO	*P. abies*	MG857101	MG857097	this study
*S. hyperborea*	Spirin 11066 (O)	NO	*P. abies*	MG857102	MG857096	this study

Specimens examined (sequenced specimens are marked by asterisk)

*Mycostilla vermiformis*. France. Aveyron (lectotypes of *Heterochaetella crystallina* and *H. dubia* var. *psilochaeta*, see below). Norway. Vestfold: Larvik, Jordstøyp i Kvelde, *Picea abies*, 15.IX.2016 *Spirin 11096* (infected by *Spiculogloea minuta*) (O), Vemannsås, *P. abies*, 30.IX.2018 *Spirin 12532* (O). Telemark: Bamble, Rogn, *P. abies*, 3.XI.2017 *Spirin 11800* (O). Møre og Romsdal: Aure, Hermundslia, *Pinus sylvestris*, 20.III.2004 Oldervik 135.04* (O F188059), Lia, *P. sylvestris*, 15.VIII.2004 *Oldervik 470.04* (O F188160); Nesset, Eikesdalen, *P. abies* (?), 27.IX.2008 *Læssøe* (O F69226), *P. sylvestris*, 28.IX.2017 Spirin 11621* (O). Sør-Trøndelag: Hemne, Gammelsetra, *P. sylvestris*, 24.X.2004 *Oldervik 620.04* (O F187964). Nordland: Grane, Litltuva, *P. abies*, 6.IX.2011 *Svantesson 86* (O F253602), Hattfjelldal, *P. abies*, 5.IX.2011 *Svantesson 89* (O). Russia. Nizhny Novgorod Reg.: Lukoyanov Dist., Panzelka, *P. abies*, 2.VIII.2017 Spirin 11330* (H). Sweden. Norrbotten: Boden, Blåkölen, *P. abies*, 19.IX.2010 *J. Nordén 6759* (O).

*Stypella papillata*. Brazil. Santa Catarina: Blumenau, no collecting date, *Möller 24* (HBG) (designated here as a lectotype, MBT383478).

*Stypellopsis farlowii*. Russia. Khabarovsk Reg.: Solnechnyi Dist., Igdomi, *Picea ajanensis*, 3.IX.2016 *Spirin 10901* (H). USA. New Hampshire (holotype, see below). Tennessee: Blount Co., Great Smokey Mts. Nat. Park, *Pinus* sp., 28.IX.2015 *Miettinen 19508.1* (H); Cocke Co.: Cosby, decayed wood, 16.VII.2004 Larsson 12337* (GB, H); Sevier Co.: Great Smokey Mts. Nat. Park, *Tsuga canadensis*, 30.IX.2015 *Miettinen 19579, 19580.2* (H). Washington: Jefferson Co., Morgan Crossing, *Tsuga heterophylla*, 7.X.2014 Spirin 8254* (H).

*Stypellopsis hyperborea*. Finland. Pohjois-Karjala: Lieksa, Louhivaara, *P. sylvestris*, 28.VIII.2002 *Junninen 3231* (H.K.), Pankasaari, *P. sylvestris*, 22.VIII.2002 *Junninen 2967* (H.K.). Etelä-Häme: Padasjoki, Vesijako, coniferous wood, 13.IX.2001 Larsson 11751* (GB). Inarin Lappi: Inari, Kessi, *P. sylvestris*, 26.VIII.1991 *Kotiranta 8458* (H.K.). Norway. Akershus: Hurdal, Fjellsjøkampen, *P. abies*, 2.IX.1990 *Hallenberg 345/90* (O F295642); Jevnaker, *P. abies*, IX.2011 J. Nordén 9751* (O). Oppland: Sel, Sagåa Nat. Res., *P. abies*, 13.IX.2016 Spirin 11061, 11066* (holotype, see below). Hedmark: Løten, Brendkoia, *P. sylvestris*, 23.IX.1987 *Høgholen 280/87* (O F160519). Møre og Romsdal: Aure, Aure, *P. sylvestris*, 6.XI.2003 *Oldervik 564.03* (O F188249), Lia, *P. sylvestris*, 29.X.2004 *Oldervik 640.04* (O F188144), Løvika, *P. sylvestris*, 10.IX.2003 *Oldervik 452.03* (O F189387). Sør-Trøndelag: Meldal, Urvatn, *P. abies*, 1.X.1991 *Bendiksen & Høiland 64*-*142* (O F173197), Tydal, *P. abies*, 19.IX.2011 *J. Nordén 9292* (O). Nor-Trøndelag: Snåsa, *P. abies*, 27.IX.2011 *J. Nordén 9692* (O). Nordland: Hattfjelldal, *P. abies*, 8.IX.2011 *Svantesson 192* (O); Rana, Ørtfjellmoen, *P. abies*, 11.IX.1976 *Strid 753/76* (O F160518). Russia. Primorie: Krasnoarmeiskii Dist., Valinku, *P. ajanensis*, 29.VIII.2013 *Spirin 6487* (H). Sweden. Västergötland: Björketorp, Klippan, *P. sylvestris*, 19.IX.1979 *Hjortstam 11028* (O F 160517). Småland: Jönköping, Marieholmsskogen, *P. abies*, 30.X.2010 *J. Nordén 7971* (O).

## Results and discussion

**Fig. 1:**
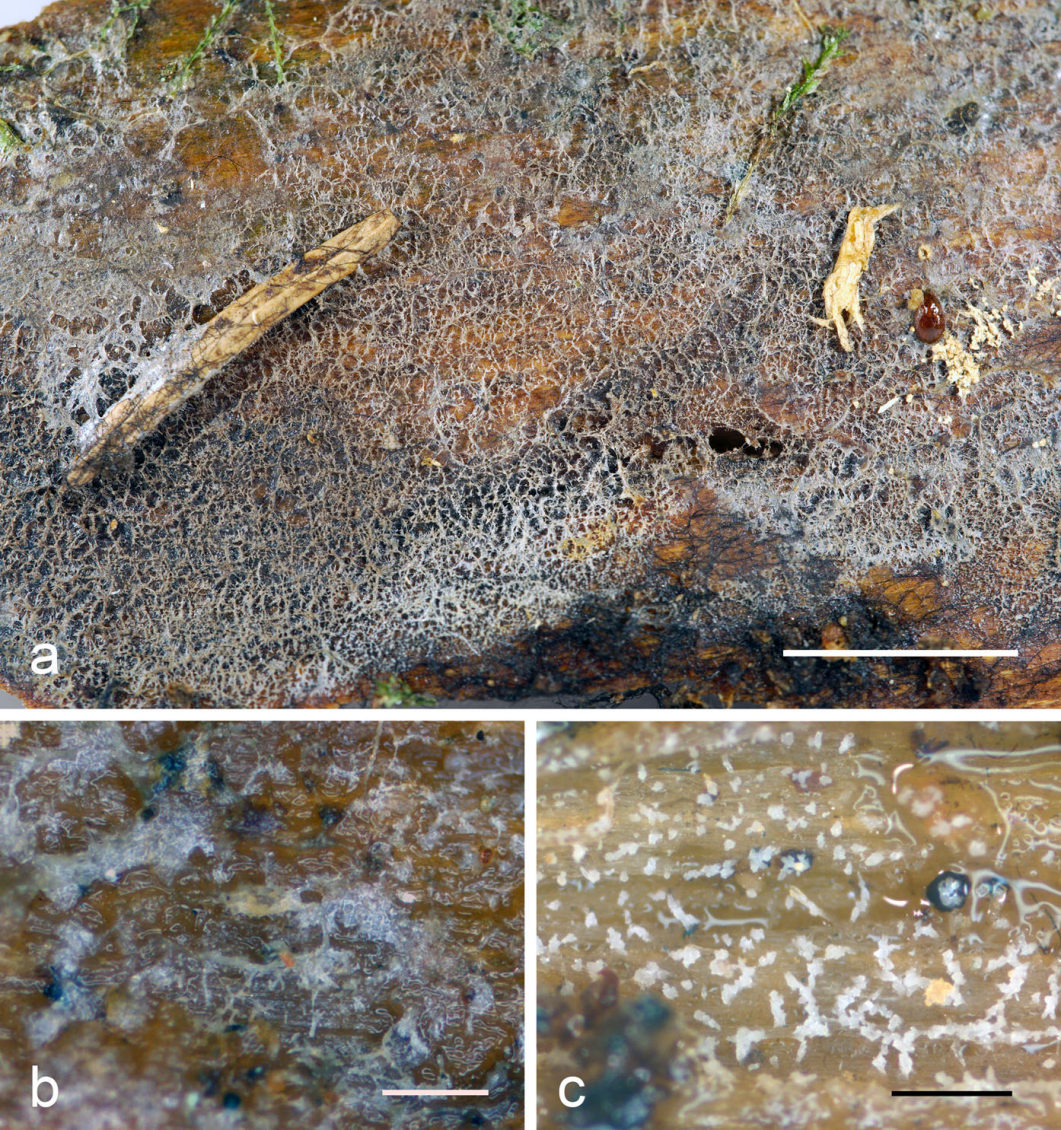
Basidiocarps of: **a***Stypellopsis hyperborea* (holotype) (scale bar = 5 mm); **b***S. farlowii* (Spirin 8254) (scale bar = 0.5 mm); **c***Mycostilla vermiformis* (Spirin 11621) (scale bar = 0.5 mm)

**Fig. 2:**
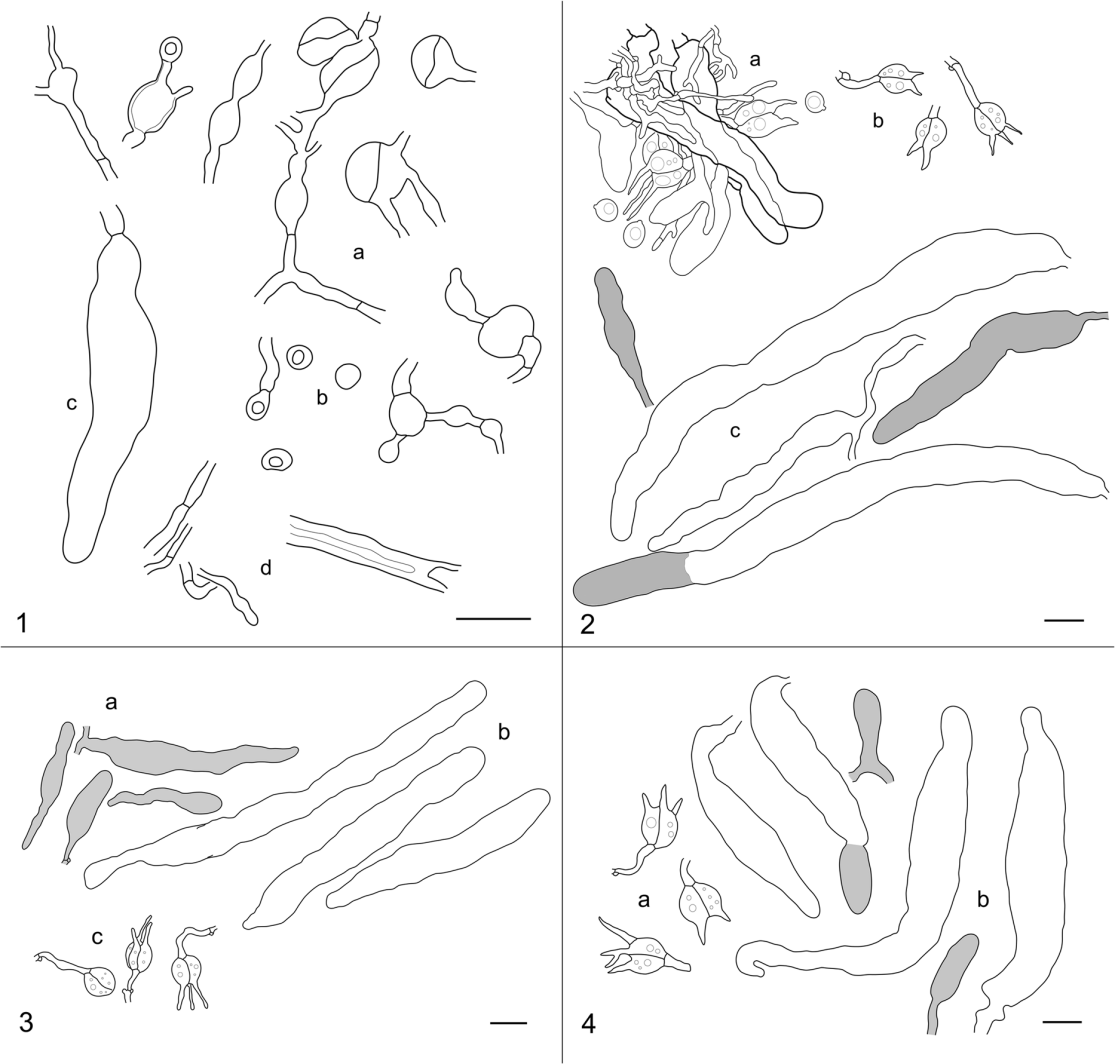
Microstructures of (**1**) *Stypella papillata* (lectotype): a – conidiophores (as observed in normal light and KOH mountant), b – conidia, c – cystidium, d – hyphae; (**2**) *Stypellopsis farlowii*: a – hymenial cells, hyphae and basidiospores (Spirin 10901), b – basidia, c – gloeocystidia and cystidia(Spirin 8254); (**3**) *Mycostilla vermiformis* (Spirin 11330): a – gloeocystidia, b – cystidia, c – basidia; (**4**) *Stypellopsis hyperborea* (holotype): a – basidia, b – gloeocystidia and cystidia. Scale bar = 10 μm

In total, 40 specimens from Europe, East Asia and North America corresponding to the current concept of *S. vermiformis* (sensu Reid 1990; Roberts 1998) were selected for morphological study. Basidiocarps are initially represented by minute, sharp-pointed, gelatinous outgrowths irregularly arranged on an extremely thin joint subiculum. These outgrowths quickly fuse together and produce compound resupinate basidiocarps of a very characteristic, reticulate appearance (observable under lens) (Fig. 1). The basidiocarp core consists of several tubular, blunt-pointed or tapering, thin-walled cystidia up to 220 μm long. These giant cystidia are often glued together and covered by sparsely arranged hyphae, basidia and occasionally by cystidia-like cells of smaller size (gloeocystidia). Basidiospores are rather small-sized, broadly ellipsoid to subglobose, repetitive, ca. 3.5–7 × 3–6 μm (Fig. 2). All studied specimens were collected from coniferous wood at advanced decay stages in temperate–boreal forests in the Northern Hemisphere.

The authentic specimen of *S. papillata* (designated as a lectotype in Specimens examined) was detected in HBG by Friedrichsen (1977) and studied by us. This fungus is widely different from *S. vermiformis*. Its fructifications are continuous, not reticulate, and up to 0.5 mm thick. Microscopically, it is characterized by slightly or distinctly thick-walled and totally clampless hyphae (as illustrated in Möller’s original figure) and a presence of globose conidiiferous cells. These cells are arranged in chains connecting with each other by hypha-like outgrowths. Walls of some conidiophores are somewhat shrunken and, if observations are made in normal light and without colouring medium, they make an impression of the inner cell septation (Fig. 2). However, using phase contrast illumination and coloured mountant (Cotton Blue) reveals it merely as an artifact. “Spores” described by Möller (1895) in the protologue of *S. papillata* are in fact broadly ellipsoid or subglobose, slightly thick-walled conidia located on short terminal projections of some conidiiferous cells. A few cystidia-like elements have been detected in *S. papillata*, too, but it is uncertain whether they belong to this fungus or another species underneath. In turn, no conidial stage has been detected in specimens of *S. vermiformis* s.l. from Northern Hemisphere, and those collections reveal regularly clamped hyphae. Two anamorphic genera so far detected in the *Auriculariales*, *Helicomyxa* R. Kirschner & Chee J. Chen and *Ovipoculum* Zhu L. Yang & R. Kirschner, also possess clamped hyphae (Kirschner and Chen 2004; Kirschner et al. 2010). The only species with clampless hyphae confirmed as a member of the *Auriculariales* is *Endoperplexa enodulosa* (Hauerslev) P. Roberts (Weiss et al. 2004b); however, it is not reminiscent of *S. papillata* either. Considering these observations, we reject the synonymy of *S. papillata* and *S. vermiformis* s.l. The identity of *S. papillata* should be re-established based on newly collected and sequenced material from the *locus classicus*.

Eight collections of *Stypella vermiformis* s.l. were selected for DNA study, and two datasets were assembled for phylogenetic analyses:

1. A nrLSU phylogeny of the *Auriculariales* (Fig. 3a). The final aligned dataset included 863 characters (including gaps). The overall topologies of the ML and BI trees were nearly congruent. Specimens of *S. vermiformis* s.l. ended up in two distantly related clades within the order:
  A. *Stypella vermiformis* s.str. clade (BS = 100, pp = 1) appeared as a sister group of *Pseudohydnum gelatinosum* although this relationship is supported by Bayesian inference only (pp = 0.95). This clade encompasses specimens collected in temperate forests of Europe. They all possess small-sized basidia and basidiospores and are thus considered conspecific with the type material of *S. vermiformis* (sensu Reid 1990 and Roberts 1998). Since *Stypella* is typified with *S. papillata* and not related to *S. vermiformis*, a new genus, *Mycostilla*, is introduced for the latter species.
  B. *Protomerulius farlowii* clade (BS = 99, pp = 1) clusters with a large group of poroid, hydnoid and corticioid species from the genera *Protomerulius* A. Möller, *Heterochaetella* (Bourdot) Bourdot & Galzin and *Hyalodon* V. Malysheva & Spirin (see further comments in Malysheva et al. 2018). This clade contains North-American specimens identical to the type specimen of *P. farlowii* Burt, as well as collections from boreal forests of North Europe. They differ from specimens in the *S. vermiformis* s.str. clade in having larger basidia and basidiospores, and somewhat longer, occasionally tapering cystidia staining brownish in KOH. A new genus, *Stypellopsis*, is described below to designate this clade.

**Fig. 3:**
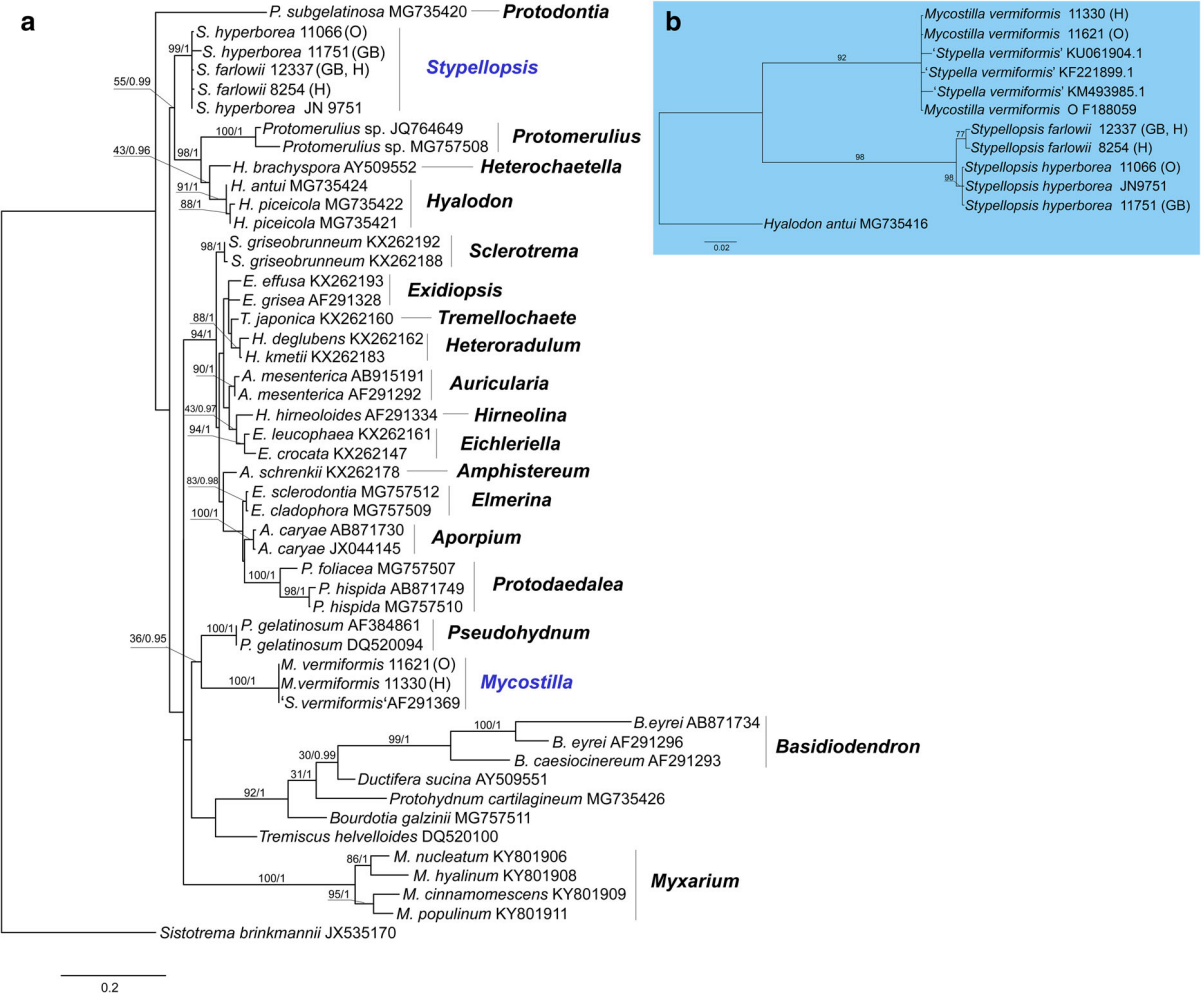
Phylogenetic relationships of lineages within the *Auriculariales*: **a** the best tree from the ML analysis of the nrLSU dataset. ML BS and Bayesian posterior probability (pp) values for internal nodes are given above the branches (BS/pp); **b** the best tree from the ML analyses of the nrITS dataset. Voucher numbers are given for newly sequenced specimens and accession numbers for additional sequences retrieved from GenBank. Scale bar shows expected changes per site
2. A nrITS phylogeny of the *S. vermiformis* complex (Fig. 3b). The final aligned dataset included 656 characters (including gaps). The ML topology shows two clades with high support (BS > 90%) corresponding to those in the nrLSU phylogeny. Whereas sequences of *M. vermiformis* are highly uniform, the *Stypellopsis* clade is split in two lineages—North American (BS = 77) and North European (BS = 98). Due to observed differences in morphology and geographic distribution, we interpret them as two species, *Stypellopsis hyperborea* (a new species from North Europe) and *Stypellopsis farlowii* (based on *Protomerulius farlowii* from North America). nrITS sequences of these species show a very little inner variation and they are constantly different in six base pairs from each other. Differences in ITS region between most closely related species in the *Auriculariales* may vary from five (*Eichleriella shearii*—*E. macrospora* complex) to fifteen base pairs (*Heteroradulum deglubens*—*H. kmetii* complex) (Malysheva and Spirin 2017). Therefore, we found the aforementioned genetic difference between *S. farlowii* and *S. hyperborea* sufficient enough to introduce them as separate taxa. Their descriptions are presented below.

Taxonomic changes proposed in our study are summarized in Table 2.

**Table 2:** Taxonomic changes proposed in the present study

Taxonomic synonyms of *Stypella vermiformis* (fide Donk 1966 and Reid 1974)	Accepted in the present study as
*Dacrymyces vermiformis* Berk. & Broome	*Mycostilla vermiformis* (Berk. & Broome) Spirin & V. Malysheva
*Stypella papillata* A. Möller	*Stypella papillata* A. Möller
*Heterochaetella crystallina* Bourdot	*Mycostilla vermiformis* (Berk. & Broome) Spirin & V. Malysheva
*Protomerulis farlowii* Burt (Donk 1966—with question mark)	*Stypellopsis farlowii* (Burt) Spirin & K.H. Larss.

## Taxonomy

### *Mycostilla* Spirin & V. Malysheva, gen. nov.

#### MB 826891

Basidiocarps appearing as small gelatinous outgrowths on a hardly visible joint subiculum, later fusing into reticulate compound fructifications. Hyphal structure monomitic, hyphae clamped, subicular hyphae interwoven, subhymenial hyphae ascending. Tramal cystidia tubular, slightly tapering upwards, apically blunt. Gloeocystidia often present, running more or less parallel to tramal cystidia. Cystidia of both types with hyaline content unchanging in KOH. Basidia 2–4-celled, pedunculate, 7–9 × 6–8 μm, with slender, distantly located sterigmata. Basidiospores thin-walled, subglobose, repetitive, 3.5–6 × 3–5 μm, often with one large oil drop.

Type species. *Dacrymyces vermiformis* Berk. & Broome.

**Fig. 4:**
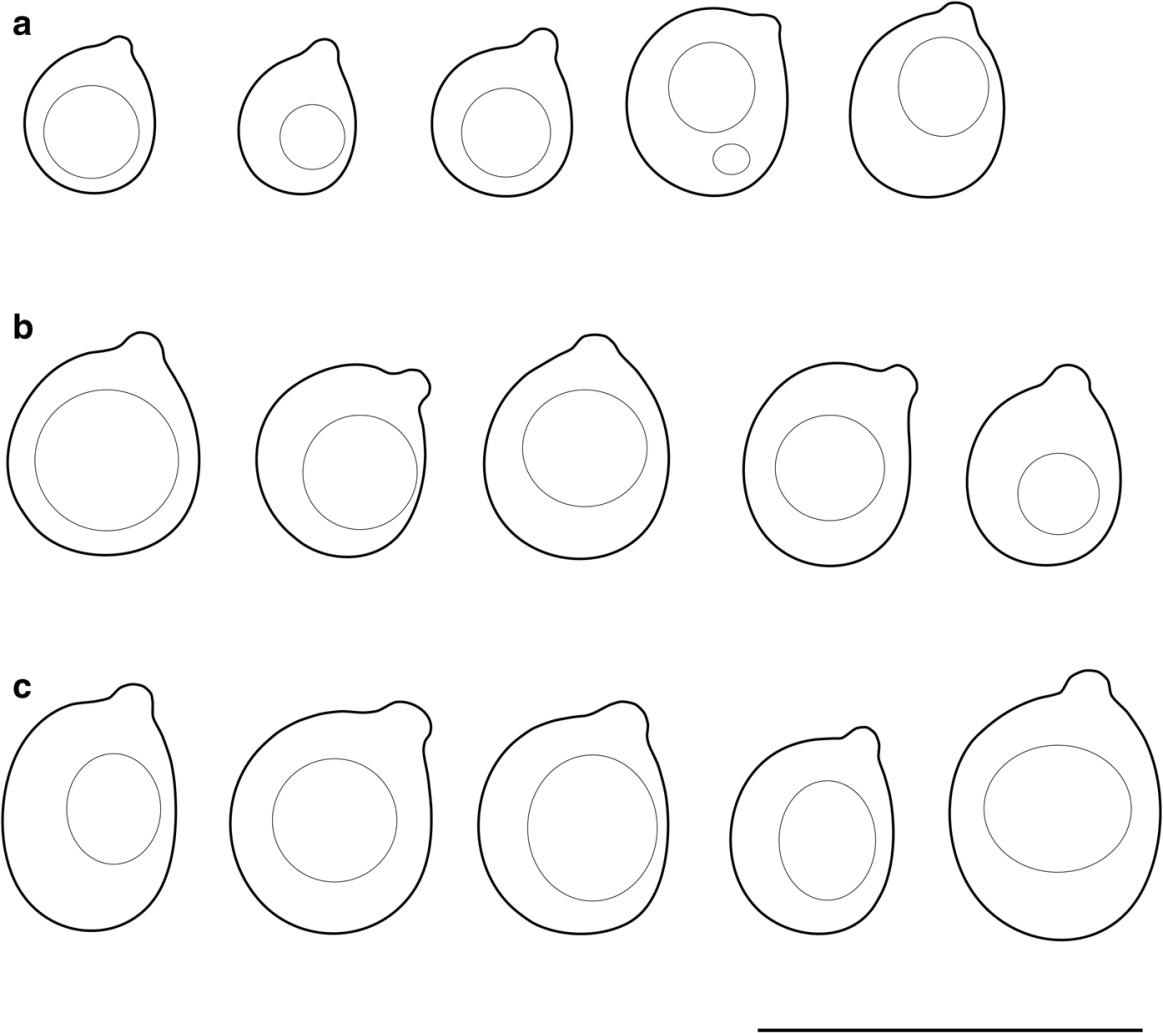
Basidiospores: **a***Mycostilla vermiformis* (Spirin 11330), **b***Stypellopsis farlowii* (Spirin 8254), **c***S. hyperborea* (holotype). Scale bar = 10 μm

*Mycostilla vermiformis* (Berk. & Broome) Spirin & V. Malysheva, comb. nov.—Figures 1, 2, 4.

≡ *Dacrymyces vermiformis* Berk. & Broome, Ann. Mag. Nat. History 1: 25, 1878. Lectotype. United Kingdom. England: Somerset, Bathford, on wood, 1.IV.1877 *Broome 404* (K(M) 47312) (selected by Reid 1974).

#### MB 826892

= *Heterochaetella crystallina* Bourdot, Trans. British Mycol. Soc. 7: 53, 1921. Lectotype. France. Aveyron: Causse Noir, *Pinus* sp., 22.XI.1914 *Galzin 16765* (herb. Bourdot 14157, PC) (designated here, MBT 384443).

= *Heterochaetella dubia* var. *psilochaeta* Bourdot & Galzin, Hyménomycètes de France: 52, 1928. Lectotype. France. Aveyron: Saint-Estève, *Juniperus* sp., XI.1910 *Galzin 7695* (herb. Bourdot 8753, PC) (designated here, MBT 384444).

= *Sebacina psilochaeta* (Bourdot & Galzin) L.S. Olive, Bull. Torrey Bot. Club 85: 89, 1958 (invalid combination, ICN Art. 41.1—basionym not indicated).

Basidiocarps gelatinous, first appearing as conical or needle-like outgrowths 0.05–0.2 mm long, partly fusing, semitranslucent, greyish or with faint pinkish or violaceous tints, later coalescent and producing reticulate compound basidiocarps 0.5–1 cm in diam. Hyphae clamped; subicular hyphae with distinct walls, 2–3.5 μm in diam., subhymenial hyphae thin-walled, 1–2 μm in diam. Tramal cystidia tubular, apically obtuse, 52–140 × 8–18 μm (n = 20/2); gloeocystidia tubular to somewhat fusiform, running parallel to tramal cystidia, 16–94 × 4–9 μm (n = 20/2). Hyphidia occasionally branched, 1–3 μm in diam. Basidia (6.8–) 7.0–8.7 (–8.8) × (5.8–) 6.1–7.6 (–7.8) μm (n = 20/2), sterigmata only rarely bifurcate, 3–16 × 1–2 μm, enucleate stalk 4–16 × 1.7–2.8 μm. Basidiospores (3.3–) 3.7–5.9 (–6.2) × (2.9–) 3.0–5.2 (–5.9) μm (n = 160/6), L = 4.28–5.22, W = 3.72–4.70, Q = 1.10–1.15.

Remarks. *Mycostilla vermiformis* is distributed in temperate forests of Europe (Svrček 1950; Hauerslev 1976; Wojewoda 1981; Reid 1990; Roberts 1998). Reid (1974, 1990) studied types of both *Dacrymyces vermiformis* and *Heterochaetella crystallina* and concluded that they were conspecific. Smaller basidia and basidiospores help to distinguish *M. vermiformis* from the similarly looking *Stypellopsis* species. Brownish colouration of cystidial content in KOH can help in identification of recent *Stypellopsis* spp. samples; this reaction is absent in all specimens of *M. vermiformis* studied by us. Attention should be paid to the age and collecting data of a specimen, nonetheless. We studied and sequenced one specimen of *M. vermiformis* (O F188059) with abnormally large basidia, 7.7–11.2 × 6.1–8.1 μm. This basidial size might point towards *Stypellopsis* spp. However, other microscopic structures of this collection, as well as ITS sequence, are identical to other specimens of *M. vermiformis*. This morphological deviation can possibly be explained as due to unusual fructification time (the specimen was collected in March).

One specimen of *M. vermiformis* from Norway was infected by *Spiculogloea minuta* P. Roberts (Spiculogloeomycetes, Pucciniomycotina), and this is the first record of this mycoparasite in the country. *Spiculogloea minuta* was originally described as a parasite of *Tulasnella violea* (Quél.) Bourdot & Galzin (Roberts 1997). Another Norwegian record (Akershus: Bærum, Kjaglidalen, 16.IX.2016, *Spirin 11125*, O) came from *T. violea*, too, and the third one from *Tulasnella deliquescens* (Juel) Juel (Oppland: Vågå, Veogjelet, 13.IX.2016, *Spirin 11081*, O). Rödel (2014) reported *S. minuta* as growing in basidiocarps of *Phanerochaete sordida* (P. Karst.) J. Erikss. & Ryvarden in Germany, and Spirin et al. (2016) detected it in *Hyphoderma argillaceum* (Bres.) Donk in European part of Russia. It is still uncertain, however, if *S. minuta* is a single species able to infect various corticioid fungi, or if we are dealing with a complex of several cryptic species.

### *Stypellopsis* Spirin & V. Malysheva, gen. nov.

#### MB 826893

Basidiocarps appearing as small gelatinous outgrowths from a hardly visible subiculum, later fusing into reticulate, compound basidiocarps. Hyphal structure monomitic, hyphae clamped, subicular hyphae embedded in a solid gelatinous matter and almost indiscernable, subhymenial hyphae ascending, some brownish in KOH. Tramal cystidia tubular, slightly or distinctly tapering upwards, apically blunt or sharpened, occasionally somewhat moniliform. Gloeocystidia often present, arising at different levels, irregularly arranged, often pleural. Some cystidia with brownish-coloured contents in KOH. Basidia 2–4-celled, pedunculate, 8–11.5 × 7–11.5 μm, with slender or rather thick, occasionally branched sterigmata. Basidiospores thin-walled, broadly ellipsoid to subglobose, repetitive, 4.5–7 × 4–6 μm, often with one large oil drop.

Type species. *Stypellopsis hyperborea* Spirin & V. Malysheva.

*Stypellopsis farlowii* (Burt) Spirin & K.H. Larss., comb. nov.—Figures 1, 2, 4.

≡ *Protomerulius farlowii* Burt, Annals Missouri Bot. Gdn. 6: 176, 1919. Holotype. USA. New Hampshire: Carroll Co., Chocorua, very rotten coniferous wood, IX.1918 *Farlow 6 *+ (FH 00488287). Epitype. USA. Tennessee: Cocke Co.: Cosby, decayed wood, 16.VII.2004 *Larsson 12337* (GB) (designated here, MBT383479).

#### MB 826894

Basidiocarps gelatinous, first appearing as needle-like, then fimbriate outgrowths 0.05–0.2 mm long, partly fusing, semitranslucent, whitish or with faint violaceous tints, later coalescent and producing reticulate compound basidiocarps 0.5–1 cm in diam. Hyphae clamped, thin-walled, 1.5–3 μm in diam., some brownish in KOH. Tramal cystidia tubular, tapering to somewhat moniliform or obtuse, some brownish in KOH, 60–220 × 8–18 μm (n = 25/3); hymenial cystidia (gloeocystidia) clavate to tapering, some pleural, irregularly arranged, 16–44 × 4–8 μm. Hyphidia occasionally branched, 0.5–3 μm in diam. Basidia (7.3–) 8.0–10.3 (–10.6) × (7.0–) 7.1–8.8 (–9.1) μm, sterigmata only rarely branched, 3–11 × 1–2 μm, enucleate stalk 3–16 × 2–3 μm. Basidiospores (4.3–) 4.6–6.1 (–6.2) × (4.0–) 4.1–5.2 (–5.3) μm (n = 150/5), L = 5.29–5.38, W = 4.62–4.84, Q = 1.10–1.17.

Remarks. Burt (1919) mistook shallow pits of a compound, reticulate basidiocarp of this species for minute pores and thus described it as a poroid fungus (see also Martin 1952). We made recent collections of this species from North America, which are identical to the type material as described by Burt (1919). Due to scantiness of the *P. farlowii* holotype, we decided to designate an epitype here. Basidiospores of *Stypellopsis farlowii* are on average larger than in *M. vermiformis* and smaller than in *S. hyperborea*. Luck-Allen (1960) reported a number of collections from Northeastern USA and Canada identified as *Stypella papillata*. In fact, they may belong to *Stypellopsis farlowii*.

*Stypellopsis hyperborea* Spirin & V. Malysheva, sp. nov.—Figure 1, 2, 4.

Holotype. Norway. Oppland: Sel, Sagåa Nat. Res., *Picea abies*, 13.IX.2016 *Spirin 11066* (O).

Etymology. Hyperboreus (Lat., adj.)—northern.

#### MB 826895

Basidiocarps gelatinous, first appearing as conical or needle-like outgrowths 0.05–0.3 mm long, then fimbriate, partly fusing, semitranslucent, whitish or with faint violaceous tints, later coalescent and producing reticulate compound basidiocarps 0.5–4 cm in diam. Hyphae clamped, thin-walled, 1–2.5 μm in diam., some brownish in KOH. Tramal cystidia tubular, tapering to somewhat moniliform or obtuse, some brownish in KOH, 60–210 × 9–17.5 μm (n = 32/3); hymenial cystidia (gloeocystidia) clavate to tapering, some pleural, irregularly arranged, 14–32 × 4–12 μm. Hyphidia occasionally branched, 0.5–2 μm in diam. Basidia (9.2–) 9.3–11.3 (–11.8) × (7.8–) 8.0–11.4 (–12.1) μm, sterigmata occasionally branched, 5–19 × 1.8–3 μm, enucleate stalk 7–30 × 2–3.5 μm. Basidiospores (4.8–) 5.1–7.0 (–7.1) × (4.0–) 4.1–5.9 (–6.2) μm (n = 200/7), L = 5.77–6.07, W = 4.89–5.21, Q = 1.14–1.19.

*RemarksStypellopsis hyperborea* possesses the largest basidiospores of the species complex. It seems to be a truly boreal species distributed in coniferous forests of North Europe. The description and microscopic drawing of *Stypella vermiformis* by Strid (1986) refer to this species.
